# Management of metastatic malignant thymoma with advanced radiation and chemotherapy techniques: report of a rare case

**DOI:** 10.1186/s12957-014-0427-z

**Published:** 2015-02-25

**Authors:** Mark A D’Andrea, G Kesava Reddy

**Affiliations:** University Cancer and Diagnostic Centers, 12811 Beamer Road, Houston, TX 77089 USA

**Keywords:** Intensity modulated radiation therapy, Image-guided radiation therapy, Mediastinal mass, Neoadjuvant chemotherapy, Surgical resection, Thymic neoplasm

## Abstract

Malignant thymomas are rare epithelial neoplasms of the anterior superior mediastinum that are typically invasive in nature and have a higher risk of relapse that may ultimately lead to death. Here we report a case of an advanced malignant thymoma that was successfully treated with neoadjuvant chemotherapy followed by surgical resection and subsequently with advanced and novel radiation therapy techniques. A 65-year-old male was diagnosed with a stage IV malignant thymoma with multiple metastatic lesions involving the left peripheral lung and pericardium. Initial neoadjuvant chemotherapy with a cisplatin-based regimen resulted in a partial response allowing the inoperable tumor to become operable. Following surgical resection of the residual disease, the tumor recurred within a year. The patient then underwent a course of targeted three-dimensional intensity modulated radiation therapy (IMRT) and image-guided radiation therapy (IGRT). Five years after radiation therapy, the localized soft tissue thickening at the left upper lung anterior pleural space had resolved. Seven years after radiation therapy the tumor mass had completely resolved. No recurrences were seen and the patient is well even 8 years after IMRT/IGRT with a favorable outcome. Chemotherapy with targeted three-dimensional IMRT/IGRT should be considered the primary modality for the management of advanced malignant thymoma patients.

## Background

Thymomas are rare epithelial neoplasms arising from the thymus gland and account for nearly all primary malignancies of the anterior superior mediastinum [1-3]. The term thymoma describes neoplasms that show no overt atypia of the epithelial component. The exact cause of thymomas remains unknown. These are typically slow-growing tumors that manifest themselves by local extension and when metastatic, the lesions are generally confined to the pleura, pericardium, or diaphragm [4-6]. The incidence of thymomas is estimated to be 1.3 cases per million population in the United States [7,8].

In general, thymomas are indolent tumors with a tendency toward local recurrence rather than metastasis. Thymic carcinomas, however, are typically invasive, with a higher risk of relapse and ultimately lead to death [9,10]. Thymic carcinomas are rare and have been reported to account for only 0.06% of all thymic neoplasms [11]. Thymoma patients more often present with metastatic disease, with a 5-year survival of 30 to 50% [12]. The management of inoperable advanced malignant thymoma is difficult. The optimal treatment of malignant thymoma depends upon the stage and extent of disease and includes a combination of surgical resection, chemotherapy, and radiotherapy. However, no clinical data from large randomized trials is available to guide the treatment, given the rarity of advanced malignant thymomas.

Various case series and small prospective trials have shown the clinical effectiveness of chemotherapy with multimodality regimens in the management of advanced malignant thymomas. Multi-agent chemotherapy is used neoadjuvantly to downstage the tumor rendering inoperable carcinoma operable or as palliative treatment to extend the patients’ life and improve their quality of life. Specifically, platinum-based chemotherapy using cisplatin in combination with vincristine, doxorubicin and etoposide have been shown to render inoperable invasive thymomas operable tumors [13]. Many case series and small studies have reported > 50% response rates with cisplatin-based chemotherapy and this has now become the standard of care for inoperable or metastatic malignant thymomas.

In this report, we describe a challenging case of a recurrent advanced malignant thymoma complicated by the presence of metastatic lesions in the lung and pericardium. Specifically, we present our experience in managing recurrent advanced malignant thymomas using multi-agent chemotherapy with cisplatin, adriamycin, vincristine, and cytoxan followed by surgical resection, intensity modulated radiation therapy (IMRT), and image-guided radiation therapy (IGRT). IMRT is an advanced mode of high-precision radiotherapy that uses computer-controlled multiple small radiation beams of varying intensities to deliver precise radiation doses to a malignant tumor or specific areas within the tumor. By incorporating three-dimensional computed tomography (CT) imaging technology, IMRT allows the radiation dose to conform more precisely to the three-dimensional shape of the tumor while modulating the intensity of the radiation beam and minimizing its dose to those organs and tissues unaffected by the cancer. IGRT uses a variety of two-dimensional, three-dimensional and four-dimensional imaging techniques that improve the precision and accuracy of the delivery of radiation dose to the targeted tumor tissue while minimizing the dose to the surrounding normal tissue during the course of radiation therapy.

## Case presentation

A 65-year old Hispanic male presented with complaints of chest pain and heaviness prior to the diagnosis of his disease. The patient had a history of tobacco use but had stopped smoking 20 years earlier. The patient had been otherwise healthy all of his life. Approximately 4 to 6 weeks prior to his diagnosis, he experienced an episode of chest pain and some mild shortness of breath. Initially, the patient underwent a routine chest X-ray and CT scan diagnostic procedures. Subsequently, a thorascopic biopsy was performed, and using H & E staining, tissue histology was carried out to detect the tumor development. In addition, the patient underwent a mediastinoscopy, thoracoscopy, and fine-needle biopsy to confirm the diagnosis of his disease.

The diagnostic evaluation revealed the presence of a stage IV malignant thymoma with multiple metastatic lesions involving the left peripheral lung and pericardium. Despite the presence of metastatic disease, these lesions are still oftentimes known to be amenable to surgical resection following treatment with induction chemotherapy. Therefore, to down- stage the tumor and to improve the patient’s chance of increased surgical resectability, neoadjuvant chemotherapy was initially employed. Since platinum with an anthracycline-based triplet or quartet regimen is currently the consensus treatment for malignant thymoma [14-17], this was chosen as the neoadjuvant chemotherapy in this patient. More specifically, the patient was initially administered five cycles of quartet regimen consisting of cisplatin, adriamycin, vincristine, and cytoxan. Subsequently, the patient received four cycles of cisplatin, adriamycin, and cytoxan followed by surgical resection of his residual disease. Despite the treatment with neoadjuvant chemotherapy and subsequent surgical resection the patient experienced recurrent chest disease within a year.

**Figure 1:**
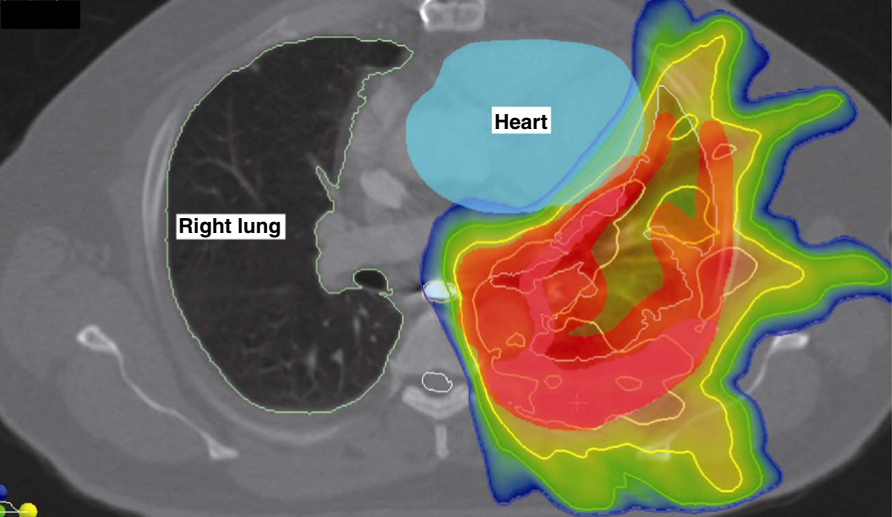
**Targeted three-dimensional intensity modulated radiation therapy and image-guided radiation therapy (IMRT/IGRT) plan depicting the high dose areas of radiation treatment in the lung pleura and chest wall of the thymoma patient.** Color changes from red to light yellow to greenish blue indicate the radiation dose from highest (in the central area) to the lowest (in the peripheral area) while adequately covering the pericardium and minimizing the radiation effect on the heart.

In attempt to salvage this patient, the patient underwent a course of post-operative and post-chemotherapy IMRT/IGRT-based three-dimensional radiation therapy to a dose of 7,440 cGy to the left chest wall, mediastinum, and left pericardium (Figure 1). The patient was monitored for safety and efficacy using CT scans and a MUltiple Gated Acquisition (MUGA) scan prior to and after his chemoradiation therapy.

### Results

**Figure 2:**
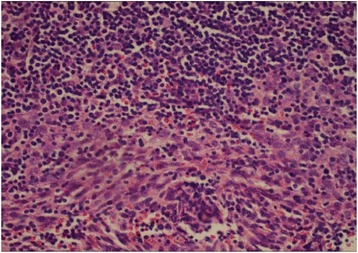
**H & E stain shows the histology of the malignant thymoma.** This was evidenced by the presence of a mixture of plump epithelial cells with both vesicular nuclei and distinct nucleoli and small lymphocytes (magnification = 400X).

Initial findings from the thorascopic biopsy indicated that the patient had multiple pleural-based masses with an associated small left pleural effusion. Assessment of tissue histology using H & E staining revealed a mixture of plum epithelial cells with both vesicular nuclei and distinct nucleoli and small lymphocytes indicating the presence of malignant thymoma (Figure 2). Following mediastinoscopy and later a thoracoscopy, the patient was provisionally suspected of having lymphoma; however, the needle biopsy assessment showed lymph with aggregates that were negative for lymphoma. Subsequent diagnostic studies confirmed the presence of a stage IV malignant thymoma. Further assessment using a multi-slice CT scan indicated that the patient had multiple metastatic lesions involving his left peripheral lung and pericardium. In addition, the chest CT imaging revealed several mass structures with the largest size being 3.4 × 6.7 cm in the left hemithorax. Furthermore, there were also multiple mediastinal masses specifically located in the anterior mediastinal region with the largest mass size measuring 3.2 cm.

The initial five cycles of neoadjuvant chemotherapy with cisplatin, adriamycin, vincristine, and cytoxan was well tolerated by the patient. However, this induction therapy only resulted in reduction in the size of the tumor masses. However, a partial response was observed after the subsequent chemotherapy with four cycles of cisplatin, adriamycin, and cytoxan. The patient then underwent surgical resection of his residual disease. Despite treatment with chemotherapy and surgical resection, the patient experienced recurrent chest disease within a year. An attempt was then made to salvage the patient and he was treated with radiation therapy.

**Figure 3:**
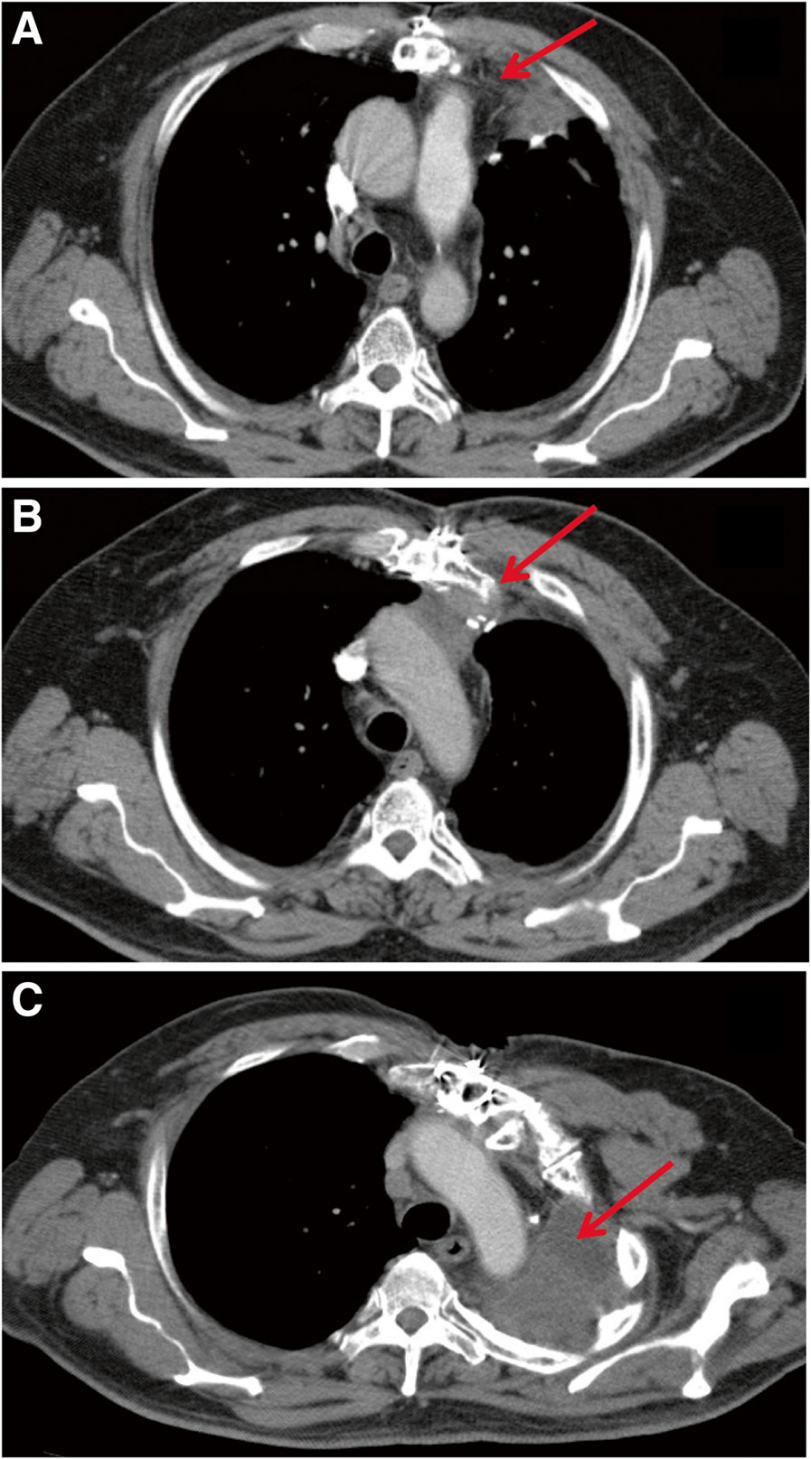
**Time-lapse computer tomography (CT) imaging of the chest of the patient with thymoma. (A)** Initial CT scan of the chest prior to targeted three-dimensional intensity modulated radiation therapy and image-guided radiation therapy (IMRT/IGRT) demonstrated a 3.7 x 3.0-cm anterior mediastinal mass. **(B)** The CT scan of the chest 5 years after post-operative chemotherapy and targeted three-dimensional IMRT/IGRT shows that the localized soft tissue thickening at the left upper lung anterior pleural space has resolved. **(C)** The CT scan of the chest 7 years after post-operative chemotherapy and targeted three-dimensional IMRT/IGRT shows that there is a moderate dependent pleural thickening at the left lung base. There is complete left lung atelectasis, but the previously seen pleural disease had resolved.

A pre-radiation therapy chest CT imaging revealed several mass structures with the largest size measuring 3.7 × 3.0 cm in the left hemithorax (Figure 3A). In addition, there were also multiple mediastinal masses located in the anterior mediastinal region with the largest mass size measuring 3.2 cm. To control the disease, the patient then underwent a course of post-operative and post-chemotherapy definitive IMRT/IGRT-based three-dimensional radiation therapy to a dose of 7,440 cGy to the left chest wall, mediastinum, and left pericardium. A post-therapy chest CT imaging showed the presence of only small sub-centimeter right middle lobe pulmonary nodules with no discrete soft tissue mass or malignancy. Follow-up CT imaging of the chest 5 years after the patient’s chemoradiation therapy, revealed that the localized soft tissue thickening at the left upper lung anterior pleural space had resolved (Figure 3B). Seven years post-chemoradiation therapy, CT imaging of the chest also showed a resolving tumor mass and postsurgical and post-radiation changes and fibrosis (Figure 3C). The patient's post-radiation MUGA scan of the heart showed that the left ventricular ejection fraction was within the expected normal value of 61% 8 years after definitive three-dimensional IMRT/IGRT.

### Discussion

Malignant thymomas are rare human neoplasms accounting for less than 0.5% of all malignancies. Due to their rarity, knowledge of the optimal treatment regarding these tumors is based on case reports or small retrospective series. Management of thymic carcinoma depends upon the clinical stage of the disease. Currently, surgical resection remains the mainstay of treatment for localized thymic malignancies [18]. However, the complete resection of the tumor is particularly important in the management of thymic carcinoma [19]. The resectability for stages I, II, III and IV of thymic carcinoma has been 100%, 43 to 100%, 0 to 85%, and 0 to 42%, respectively [20]. A study by Kondo *et al.* [21] has shown that the 5-year survival rate is 66.9%, 29.8%, and 19.4% for completely resected, incompletely resected, and non-surgery groups, respectively.

In general, extensive mediastinal or lung invasion or metastasis is a frequent finding in most newly diagnosed patients. Despite complete surgical resection, over 50% of patients with advanced disease experience recurrence [21]. Consequently, treatment with multiple modalities such as repeat surgical resection, chemotherapy, and radiotherapy has been attempted but without general consensus on the optimal approach [22]. It is known that most thymic tumors are chemo- and radio-sensitive [23-25] and, thus, a multimodality treatment that integrates surgical resection with chemo- and radiotherapy has been advocated for advanced stages with the aim to improve both local and distal control of the disease and prolong survival [26-28]. In addition, preoperative (induction or neoadjuvant) chemotherapy has been successfully used to down-stage unresectable tumors for surgical resection and to prevent local and systemic recurrences [24,27,29-37].

The present patient was diagnosed as having an advanced stage IV malignant thymoma with multiple metastatic lesions involving the left peripheral lung and pericardium. Therefore, induction chemotherapy was employed, followed by surgery, and then post-operative definitive targeted three-dimensional IMRT/IGRT in this patient. Since cisplatin-based combination chemotherapy is effective against thymic tumors [35,38,39], cisplatin was used in combination with adriamycin, vincristine, and cytoxan as induction chemotherapy to down-stage the tumor and to improve the surgical resectability of the patient’s unresectable tumor. This initial induction chemotherapy was found to be effective in reducing the sizes of the masses with an acceptable safety and patient tolerability; however, residual tumor was still present. Additional cycles of induction chemotherapy with cisplatin, adriamycin, and cytoxan yielded a partial response and the residual disease was managed with surgical resection.

Little has been reported on the role of advanced radiation therapy in the management of malignant thymoma. Until recently, no literature has demonstrated the superiority of one radiation therapy method over another. Despite the sensitivity of thymoma to radiation, the best use of radiotherapy remains controversial. Moreover, there remains no consensus on whether or not adjuvant radiation is of any benefit in completely resected thymoma. However, in this case study, we have demonstrated that by using targeted three-dimensional IMRT/IGRT, we were able to address the patient’s extensive recurrent disease and treat critical structures that were involved by the disease. In addition, our treatment approach spared the patient from any long-term detrimental side effects, especially to his myocardium and other critical organs.

## Conclusion

Multimodality therapy involving both neoadjuvant and post-operative chemotherapy in conjunction with three-dimensional well-targeted IMRT/IGRT appears to increase the success rates of complete resection and improve survival in patients with metastasized thymic carcinoma. Neoadjuvant chemotherapy with cisplatin, adriamycin, vincristine, and cytoxan resulted in a partial response allowing the inoperable tumor to become operable in this patient. Following surgical resection, targeted three-dimensional IMRT/IGRT was well tolerated without any cardiac toxicity. Targeted three-dimensional IMRT/IGRT proved to be clinically effective resulting in no serious adverse events and more importantly achieving a complete response of the tumor leading to long-term tumor control and increased survival of the patient with minimal compromise in his quality of life.

## Consent

A written informed consent was obtained from the patient for publication of this case report and any accompanying images. A copy of the written consent is available for review.

## Abbreviations

CT: Computed tomography
IMRT: Intensity modulated radiation therapy
IGRT: Image guided radiation therapy
MUGA: Multiple gated acquisition
